# Inferring causal gene regulatory network *via* GreyNet: From dynamic grey association to causation

**DOI:** 10.3389/fbioe.2022.954610

**Published:** 2022-09-27

**Authors:** Guangyi Chen, Zhi-Ping Liu

**Affiliations:** ^1^ Department of Biomedical Engineering, School of Control Science and Engineering, Shandong University, Jinan, Shandong, China; ^2^ Center for Intelligent Medicine, Shandong University, Jinan, Shandong, China

**Keywords:** gene regulatory network inference, dynamic grey association, adaptive sliding window, causation, machine learning

## Abstract

Gene regulatory network (GRN) provides abundant information on gene interactions, which contributes to demonstrating pathology, predicting clinical outcomes, and identifying drug targets. Existing high-throughput experiments provide rich time-series gene expression data to reconstruct the GRN to further gain insights into the mechanism of organisms responding to external stimuli. Numerous machine-learning methods have been proposed to infer gene regulatory networks. Nevertheless, machine learning, especially deep learning, is generally a “black box,” which lacks interpretability. The causality has not been well recognized in GRN inference procedures. In this article, we introduce grey theory integrated with the adaptive sliding window technique to flexibly capture instant gene–gene interactions in the uncertain regulatory system. Then, we incorporate generalized multivariate Granger causality regression methods to transform the dynamic grey association into causation to generate directional regulatory links. We evaluate our model on the DREAM4 *in silico* benchmark dataset and real-world hepatocellular carcinoma (HCC) time-series data. We achieved competitive results on the DREAM4 compared with other state-of-the-art algorithms and gained meaningful GRN structure on HCC data respectively.

## 1 Introduction

The gene regulatory network plays a central role in understanding the mechanisms of gene expression regulation, complex diseases, and cellular heterogeneity (Xiang et al., 2019; Fang et al., 2020; Liao et al., 2022). Compared to the genomes between human *Homo sapiens* and yeast *Saccharomyces cerevisiae*, we can conclude that the complexity in life does not result from the number of genes, but the essence and dynamics of the interactions between genes (Huynh-Thu and Sanguinetti, 2019; Freyre-González et al., 2022; Jansen et al., 2022), i.e., gene regulatory network.

Diverse methods have been proposed to infer gene regulatory networks. Recently, the emergence and rise of machine-learning methods to infer gene regulatory networks have dated back to GENIE3 (Vân et al., 2010), which won the DERAM4 (Dialog on Reverse Engineering Assessment and Methods) *in silico* multifactorial challenge. Then, Jump3 (Huynh-Thu and Sanguinetti, 2015) was proposed to learn the promoter state of the target gene from candidate regulators based on the decision tree. SWING (Finkle et al., 2018) introduced sliding windows to address heterogeneous time delays in the network structure inference. To further improve the inference accuracy, BTNET (Sungjoon et al., 2018) and BiXGBoost (Zheng et al., 2018) transformed the random forest into gradient boosting algorithms. Regularization-based regression introduced different constraints for *de novo* GRN reconstruction (Phan and Rosemary, 2018; Ghosh Roy et al., 2020; Zhang et al., 2021). BETS applied bootstrap elastic net regression based on Granger causality to infer the GRN (Lu et al., 2021). Recurrent neural network (RNN) was utilized to model gene interactions due to the superior capability of tracking complicated temporal behaviors in the real underlying regulatory system (Cheng et al., 2011; Biswas and Acharyya, 2018). Although machine-learning methods achieved great success, the internal procedures are unknown to us or they are known but hard to be understood by observers (Guidotti et al., 2018).

In this article, we propose an interpretable machine-learning method named GreyNet, i.e., dynamic grey association and regression, to infer the gene regulatory network from time-course gene expression data. We first apply dynamic grey association to model intricated underlying the regulatory system. Different from the static grey association, we assimilate the adaptive sliding window technique to conduct dynamic analysis which can better capture instant interactions over time. The dynamic grey association takes advantage of local temporal information to search for candidate regulators. Then, we embed the Granger causality framework (Arnold et al., 2007; Finkle et al., 2018; Li et al., 2020) based on regression models which can find causal and directional regulatory links. Through this hybrid strategy, GreyNet can better model gene interactions in real scenarios and is easier to be understood in network inference.

## 2 Materials and methods

**FIGURE 1:**
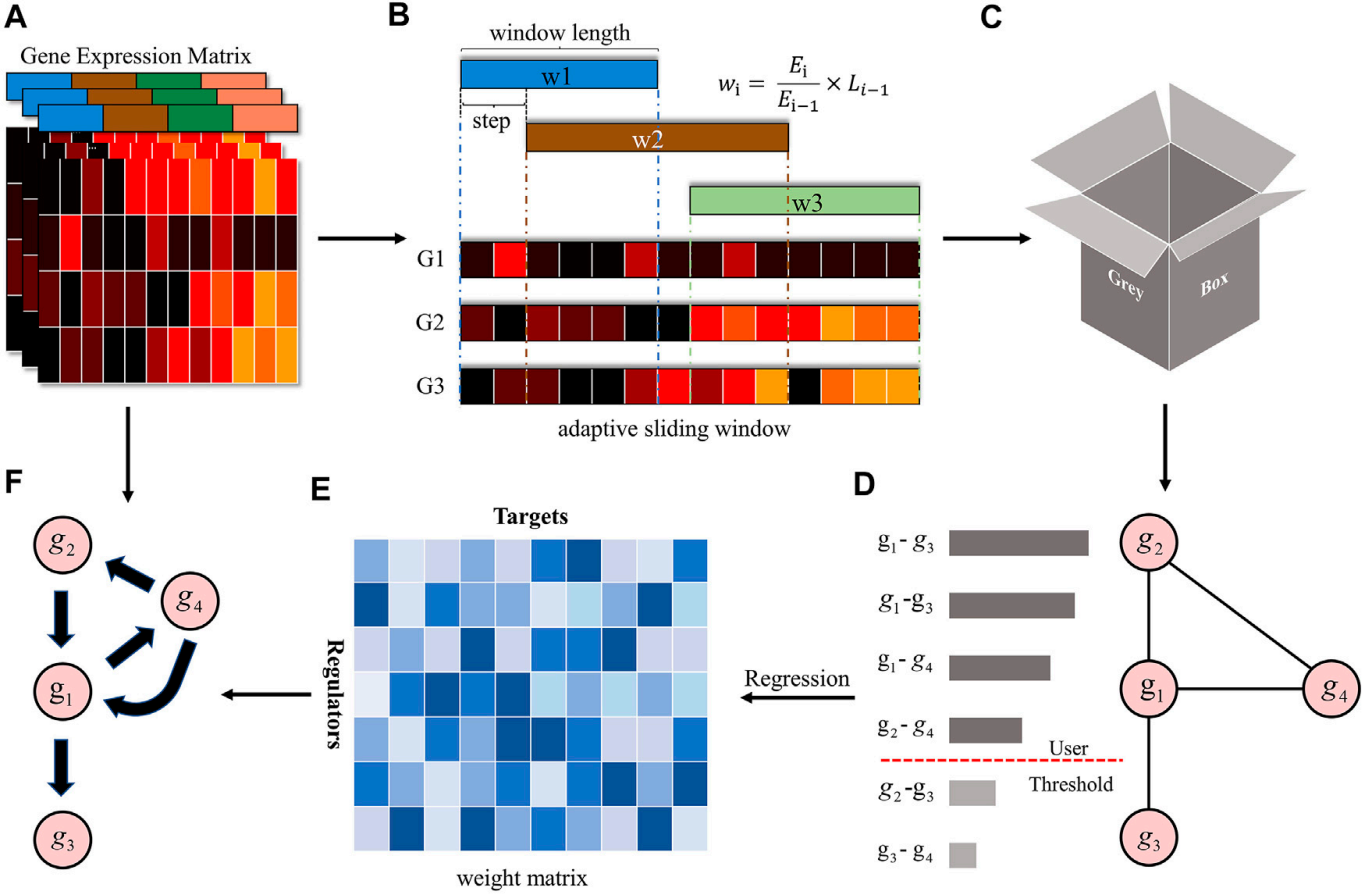
The overview of the GreyNet framework. **(A)** is the expression matrix of genes. **(B,C)** is the procedure of dynamic grey association. The window length is automatically adjusted by information entropy (IE). We firstly sample the time points by the sliding window. Then, we input the sampled data into grey relational analysis to get the dynamic grey association coefficient as **(D)**. **(E)** is the weight matrix generated by regression methods that transform the dynamic grey association to causal directional regulatory link as **(F)**.

In this section, we demonstrate GreyNet to reconstruct the directional graph of GRN *G* (*v*, *e*) from time-series gene expression data. Time-series can effectively disclose the dynamic interactions of genes with time (Haonan et al., 2020). As for the data *D*{*G*|*G* ∈ *R* ^*m*×*n*^}, *v* represents the nodes or vertexes and *e* represents the regulatory links between pairwise vertexes. For the edge *e*_*ij*_, it represents gene *j* under the upstream regulation of gene *i*. The arrangement of the whole gene expression time-series is as follows:

$$G\left(i,j\right)=\left|\begin{array}{cccc} g_{11} & g_{12} & \ldots & g_{1n}\\ g_{21} & g_{22} & \ldots & g_{2n}\\ \vdots & \vdots & \ddots & \vdots \\ g_{m1} & g_{m2} & \ldots & g_{mn} \end{array}\right|$$ (1)

where each column represents an instance and each row represents the value of the selected instance at a specific timestamp. The overall framework of GreyNet is illustrated in Figure 1. We will demonstrate the major steps in the following sections.

### 2.1 Dynamic grey association

The biological system is very complicated. Although we have revealed important mechanisms of gene regulation, we are still far from being fully clear about it. In the gene regulatory system, genes generally are under the regulation of various types of regulators and most of them are still unknown or unobserved (Ming et al., 2020). With poor data and limited knowledge at present, the GRN inference works on uncertain systems, namely the grey system between black and white. In other words, reconstructing the GRN is with partially known and unknown information, but we want to draw out the valuable GRN structure from observed gene expression data. In this condition, we propose the dynamic grey association to model gene interactions. The dynamic grey association consists of grey relational analysis (Deng, 1989; Sallehuddin et al., 2008; Yuansheng et al., 2019) and the adaptive sliding window (Papadimitriou et al., 2006). Under the circumstance of the relationships between two components are usually variational over time in the biological system, we integrate grey association with the adaptive sliding window which endows the capacity of flexibly tracking instant interactions in time-series data. Therefore, the dynamic grey association is much more interpretable than the “black box” and conforms to current knowledge.

We formally introduce how to obtain the dynamic grey association coefficient here. Firstly, we demonstrate the way to design the adaptive sliding window (Figure 1B). For the gene expression time-series $D\{\left\{\right.G|G\in R^{m\times n}\}$ , we take the first-order difference to get the time derivative. We select one gene as a target or reference node *y* = {*g*_1_, *g*_2_, *…*, *g*_*n*_} and the rest of the genes are comparative nodes *X*_*k*_ = {*G*_1_, *…*, *G*_*k*−1_, *G*_*k*+1_, *…*, *G*_*m*_}, where *G*_*k*_ is a vector that represents the expression data of gene *k*.

$$\nabla y=\left\{\nabla g_{1},\nabla g_{2},\ldots ,\nabla g_{n}\right\}$$ (2)

$$\nabla g_{n}=g_{n}-g_{n-1}$$ (3)

Then, we normalize the time derivative to transform it into probability by using the *softmax* function. Information entropy is a good choice to evaluate the amount of information.

$$p_{i}=softmax\left(\left|\nabla y\right|\right)=\frac{exp\left(\left|\nabla g_{i}\right|\right)}{\underset{\nabla g_{i}\in \nabla y}{\sum }exp\left(\left|\nabla g_{i}\right|\right)}$$ (4)

$$E_{i}=-\sum p_{i}\times log_{2}\left(p_{i}\right)$$ (5)

Finally, we divide the present information entropy by the previous one to obtain the window coefficient. The preceding window length multiplies the window coefficient to get the current sliding window. The window length will be adjusted by information entropy automatically.

$$L_{i}=\frac{E_{i}}{E_{i-1}}\times L_{i-1}$$ (6)

In every adaptive sliding window, we get the individual grey relational grade. Initially, the target node subtracts the comparative nodes’ corresponding elements to get the absolute value of the first-order norm residual ∇.

$$\nabla =|y\left(i\right)-x_{k}\left(i\right)|$$ (7)

From the maximum and minimum of the residual ∇, the association coefficient *r*_*i*_ is given by:

$$\xi_{k}\left(i\right)=\frac{\underset{k}{\min}\underset{i}{\min}\nabla +\rho *\underset{k}{\max}\underset{i}{\max}\nabla }{\nabla +\rho *\underset{k}{\max}\underset{i}{\max}\nabla }$$ (8)

$$r_{i}=\frac{1}{L}\overset{L}{\underset{i=1}{\sum }}\xi_{k}\left(i\right)$$ (9)

where *L* is the length of the adaptive sliding window. *ρ* is the distinguished coefficient which is positively related to distinguishing the difference (Kuo et al., 2008). Finally, we average all the sliding windows to get the dynamic grey association score *DGA*:

$$DGA=\frac{1}{n}\overset{n}{\underset{i=1}{\sum }}r_{i}$$ (10)

DGA determines the dominant factors between the multivariable and target gene based on the geometric curve. The higher the value of DGA, the higher the association between the two variables.

### 2.2 Causation

GRN represents directional causal regulatory relationships among genes (Leng et al., 2019). However, the dynamic grey association cannot depict causality for pairwise genes. In this case, we incorporate the Granger causality framework to turn the dynamic grey association into causation. The Granger causality is intuitive and defined that the past values of the cause make a larger contribution to predicting the future values of the effect than auto-regression. However, it is time-consuming and ignores the possible interactions between features. Inspired by LASSO Granger (Arnold et al., 2007), we apply multivariate regression strategies to identify the subset of features on which the feature is conditionally dependent, namely, we formulate it to a problem of feature selection, given the fact that the best estimator for the target variable is the one with the least error or the maximum gain (Arnold et al., 2007; Li et al., 2020).

In this study, we mainly introduce four regressors to infer GRN. The four regressors we selected are: bagging algorithm random forest (RF) (Friedman, 2001), gradient boosting algorithm XGBoost (Chen and Guestrin, 2016), *L*_1_-penalty LASSO (Tibshirani, 1996), and *L*_2_-penalty Ridge (Hoerl and Kennard, 1970). We regress target variable *y*_*t*_ in terms of *X* ^*lag*^. For different regressors, the objective function of the regressor and optimizing strategy are different. With regards to RF, it is similar to GENIE3 (Vân et al., 2010). The goal of the tree-based regressor is to build a fine decision tree structure in terms of splitting nodes. The criterion for measuring the quality of splitting is the mean squared error, which is equal to variance reduction. The evaluation of the objective function for the target is as:

$$I\left(\text{N}\right)=\#SVar\left(\text{N}\right)-\#S_{pt}\mathrm{Var}\left(S_{pt}\right)-\#S_{pf}\mathrm{Var}\left(S_{pf}\right)$$ (11)

where # is the number of the samples; *S* is the sample sets that reach the node *N* is a single tree; *S*_*pt*_ is the sample sets predicted true; and *S*_*pf*_ is the sample sets that predicted false. Nevertheless, the criterion of Xgboost regression is modified by:

$$\begin{array}{l} L_{\mathrm{split}}=\frac{1}{2}\left[\frac{G_{\mathrm{left}}^{2}}{H_{\mathrm{left}}+\lambda }+\frac{G_{\mathrm{right}}^{2}}{H_{\mathrm{right}}+\lambda }-\frac{G^{2}}{H+\lambda }\right]-\gamma \\ G_{\mathrm{left}}=\underset{i\in I_{\mathrm{left}}}{\sum }g_{i}\;\;H_{\mathrm{left}}=\underset{j\in I_{\mathrm{left}}}{\sum }h_{j}\\ I=I_{\mathrm{left}}\cup I_{\mathrm{right}} \end{array}$$ (12)

where *g*_*i*_ and *h*_*i*_ are the first-order and second-order gradient statistics. *λ* and *γ* are both complexity parameters. *I*_*left*_ and *I*_*right*_ are the sample sets after splitting. Regularization-based regression introduces different regularization terms to prevent overfitting and get optimal reconstruction. The objective function of regularized regression is:

$$Obj=\frac{1}{m}\overset{m}{\underset{i=1}{\sum }}{\left(y_{i}-X^{\mathrm{lag}}w\right)}^{2}+\lambda \overset{n}{\underset{j=1}{\sum }}\|w_{j}{\|}^{p}$$ (13)

where ‖*w*_*j*_‖^*p*^ is the regularization term. *λ* is the regularization coefficient. When *p* equals one or two, it represents the LASSO regression or Ridge regression.

Based on the aforementioned regression criteria, we can obtain weight matrix *w* from *w* ^*y*^ = *regressor* (*y*, *X* ^*lag*^) (as shown in Figure 1E). To confirm the causal regulatory direction, we summarize LASSO Granger as an example that other regression methods are similar to it. If x regulates y, *x* ^*t*^ ∈ *w* ^*y*^ for some *t* but *y*_*t*_∉*w* ^*x*^; if y regulates x, *y* ^*t*^ ∈ *w* ^*x*^ for some *t* but *x*_*t*_∉*w* ^*y*^; and if x and y regulate each other, then *x* ^*t*^ ∈ *w* ^*y*^ and *y* ^*t*^ ∈ *w* ^*x*^.

## 3 Results

### 3.1 Gene expression data

GreyNet focuses on time-course bulk gene expression data. Our method is not suitable for single-cell RNA-seq data due to dropout events. In the past decade, the DREAM challenge has been the standard benchmark dataset to evaluate the quality of the reconstructing algorithm (Marbach et al., 2009; Marbach et al., 2010). Therefore, we firstly validate GreyNet on the DREAM4 time-series dataset which contains two sizes of networks, size10 and size100, and each network includes five subnetworks. To further test the performance of our model, we evaluate it on a real-world hepatocellular carcinoma dataset (Yang et al., 2019a). HCC expression profiles are detected from 105 samples represented stepwise from pre-neoplastic lesions to HCC. 105 samples cover the nine development stages of HCC. To preprocess HCC data from NCBI GEO, we map probeset IDs to NCBI official gene symbols through the GEO annotation file. If one gene has multiple probeset mappings, the probeset with the maximum inter quartile expression range (IQR) is selected (Liu et al., 2014). Finally, we employ prior knowledge to separate the TF expression and target expression (Lambert, 2018) to conduct transcriptomic GRN inference by GreyNet. The detailed descriptions of the DREAM4 and HCC time-series expression data are shown in Table 1. The development stages of HCC from normal to hepatocellular carcinoma are shown in Table 2.

**TABLE 1:** The dscription of the datasets used in the experiments.

Network	#TF	#Gene	#Timepoints	#Samples
DREAM4 *in-silico* size 10	10	10	21	5
DREAM4 *in-silico* size 100	100	100	21	10
HCC	21	258	10	105

**TABLE 2:** The development stages of HCC.

Development	Notation	#Samples
Normal	N	13
Choronic Hepatitis with low grade	FL	8
Choronic Hepatitis with high grade	FH	12
Cirrhosis	CS	12
Dysplastic nodules with low garde	DL	11
Dysplastic nodules with high garde	DH	11
Early hepatocellular carcinoma	eHCC	5
Hepatecellular carcinoma (TG1)	TG1	9
Hepatecellular carcinoma (TG2)	TG2	12
Hepatecellular carcinoma (TG3)	TG3	12

### 3.2 Evaluation metrics

We mainly use two common metrics, AUROC and AUPRC, to evaluate our model. AUROC is calculated from the ROC curve, showing the trade-off between true positive rate (TPR) and false positive rate (FPR) across different thresholds. AUPRC is just the area under the PR curve, where the x-axis is Precision and the y-axis is Recall. The other measurement metrics, such as Precision, Matthews correlation coefficient (MCC), and Accuracy, are shown in Supplementary Table S1 (Tng et al., 2021; Le and Ho, 2022).

$$TPR=Recall=\frac{TP}{TP+FN}$$ (14)

$$FPR=\frac{FP}{FP+TN}$$ (15)

$$Precision=\frac{TP}{TP+FP}$$ (16)

### 3.3 Performance on DREAM4 data

**FIGURE 2:**
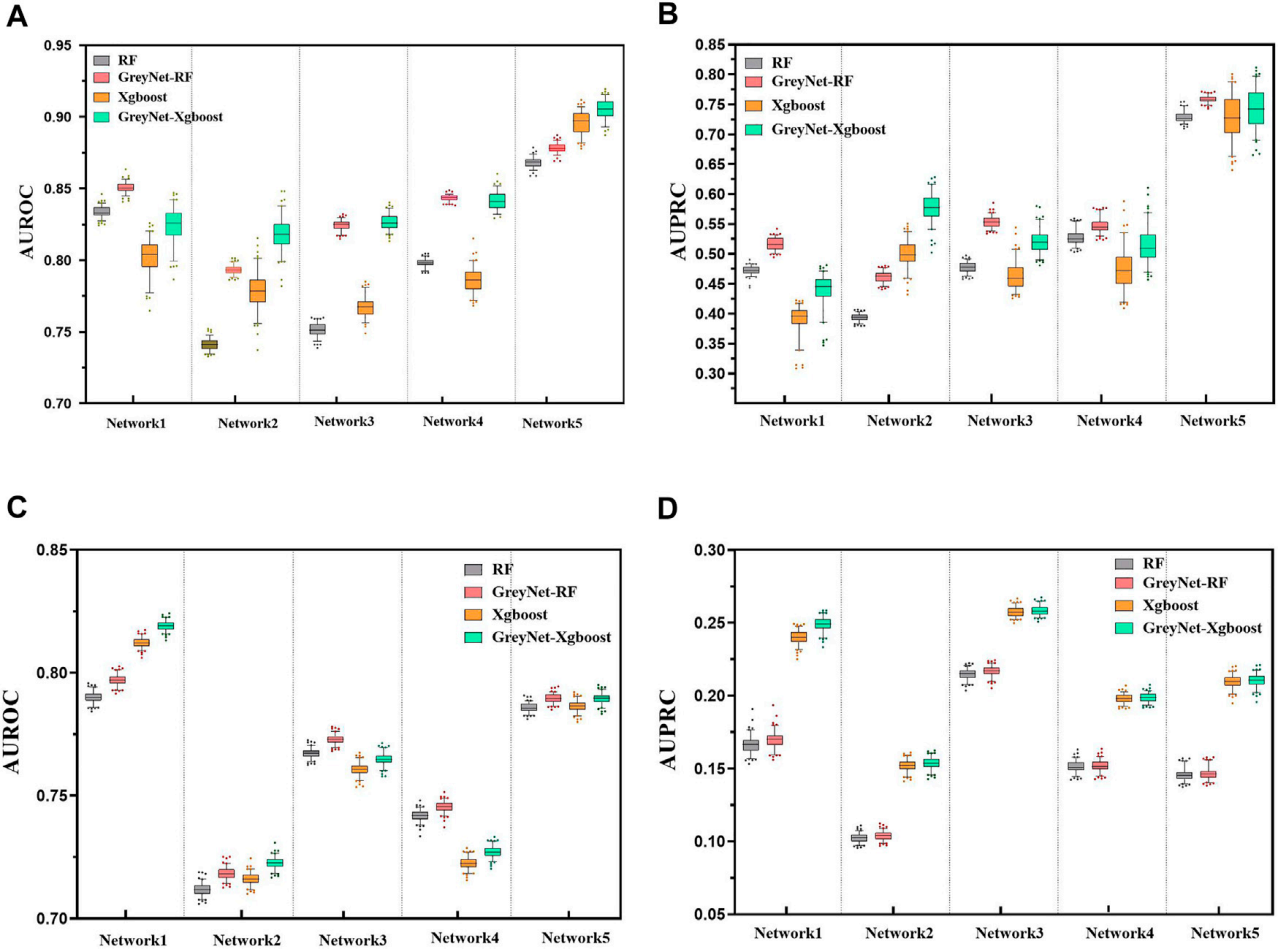
The comparison of RF, GreyNet-RF, Xgboost, and GreyNet-Xgboost on DREAM4 *insilico* datasets. **(A)** is the results of AUROC on DREAM4 size10 networks. **(B)** is the results of AUPRC on DREAM4 size10 networks. **(C)** is the results of AUROC on DREAM4 size100 networks. **(D)** is the results of AUPRC on DREAM4 size100 networks.

**FIGURE 3:**
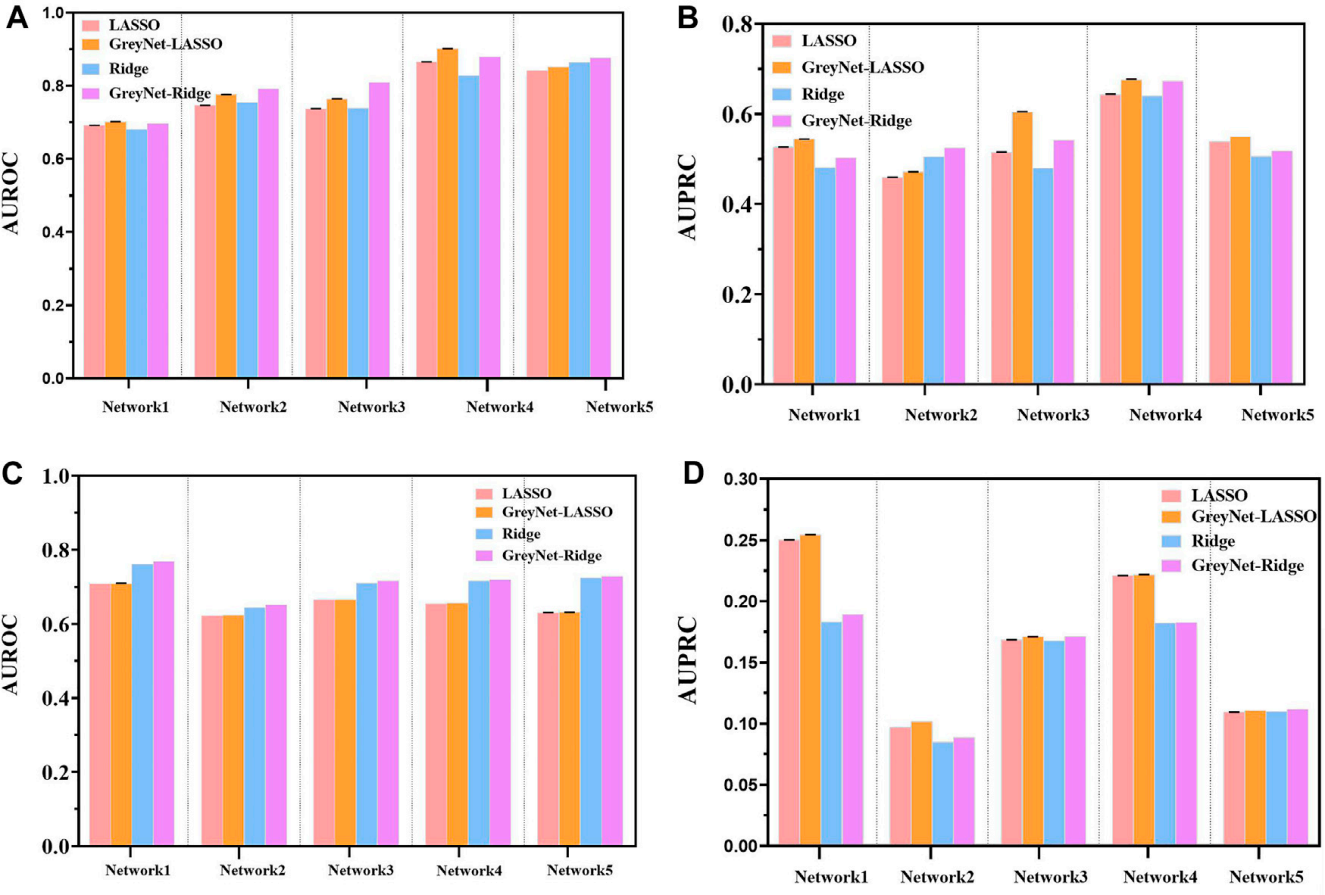
The comparison of LASSO, GreyNet-LASSO, Ridge, and GreyNet-Ridge on DREAM4 in *in silico* datasets. **(A)** is the results of AUROC on size10 networks. **(B)** is the results of AUPRC on size10 networks. **(C)** is the results of AUROC on size100 networks. **(D)** is the results of AUPRC on size100 networks.

In the DREAM4 challenge, we implement four different regression strategies on the DREAM4 dataset combined with the dynamic grey association. We select one typical model from each bagging and gradient boosting algorithm, i.e., random forest (RF) and Xgboost. Analogously, we also incorporate two different regularized regression methods *L*_1_-norm LASSO and *L*_2_-norm Ridge. For each regression method, we run them 100 times to reduce randomness. To further validate the capacity of the grey technique, we compare the four different regression methods with and without the dynamic grey association. The performances of bagging and gradient boosting comparisons are shown in Figure 2. As shown, GreyNet-RF and GreyNet-Xgboost are significantly superior to the corresponding regressors without the dynamic grey association in both AUROC [*P*_*RF*_ − *value* = 3.93–18; *P*_*Xgboost*_ − *value* = 6.57*e* − 18, Wilcoxon test] and AUPRC [*P*_*RF*_ − *value* = 3.96*e* − 18; *P*_*Xgboost*_ − *value* = 3.96*e* − 18]. In the 100 trials, two regularized regression methods have little changes in AUROC and AUPRC. The results of the two regularized regression comparisons are shown in Figure 3. From Figure 3, we can see that GreyNet-LASSO and GreyNet-Ridge significantly outperform LASSO and Ridge regressors with respect to both AUROC [*P*_*LASSO*_ − *value* = 1.86*e* − 18; *P*_*Ridge*_ − *value* = 1.55*e* − 23] and AUPRC [*P*_*LASSO*_ − *value* = 2.10*e* − 18; *P*_*Ridge*_ − *value* = 1.55*e* − 23]. Therefore, the dynamic grey association is effective and efficient to improve the GRN structure inference.

We further compare our model with other seven state-of-the-art methods, including GENIE3-lag (Huynh-Thu, 2011), Jump3 (Huynh-Thu and Sanguinetti, 2015), SWING (Finkle et al., 2018), BTNET (Sungjoon et al., 2018), BiXGBoost (Zheng et al., 2018), BETS (Lu et al., 2021), and TIGRESS(Anne-Claire et al., 2012). In the five subnetworks, the average AUROC and AUPRC of GreyNet achieve 0.854 ± 0.032, and 0.622 ± 0.108 in size10 and 0.768 ± 0.036, 0.222 ± 0.039 in size100, respectively. The detailed results of AUROC and AUPRC in each subnetwork are shown in Table 3. The ROC and PR curves of GreyNet are shown in Supplementary Figure S1. From Table 3, we can see that our model achieves the highest AUROC in networks 2, 3, and 4 of size10 among the comparing methods. Other than network 1, our model gets the highest AUPRC. In DREAM4 in-silico size100, GreyNet achieves all of the highest AUROC and AUPRC in the five networks other than AUROC in network 4. The complete performances of GreyNet with four different regression strategies are shown in Supplementary Tables S2, S3.

**TABLE 3:** The comparative results of models on DREAM4 data.

DREAM4 *in silico* size10										
Method	Network1		Network2		Network3		Network4		Network5	
	AUROC	AUPRC	AUROC	AUPRC	AUROC	AUPRC	AUROC	AUPRC	AUROC	AUPRC
GreyNet	0.839	0.479	**0.833**	**0.628**	**0.838**	**0.580**	**0.844**	**0.611**	0.917	0.812
BTNET(GB)	0.834	0.516	0.698	0.362	0.682	0.473	0.822	0.560	**0.934**	0.774
BTNET(AB)	**0.875**	0.552	0.719	0.370	0.719	0.465	0.791	0.506	0.903	0.701
SWING-RF	0.832	0.508	0.779	0.525	0.815	0.546	0.728	0.441	0.925	0.753
SWING-Dionesus	0.743	0.469	0.786	0.484	0.789	0.421	0.772	0.540	0.807	0.625
BiXGBoost	0.816	**0.573**	0.784	0.422	0.771	0.376	0.787	0.533	0.888	0.741
GENIE3-lag	0.834	0.476	0.741	0.391	0.750	0.478	0.797	0.520	0.869	0.734
Jump3	0.700	0.442	0.698	0.308	0.717	0.401	0.784	0.486	0.841	0.619
TIGRESS	0.758	0.375	0.602	0.222	0.618	0.200	0.764	0.324	0.804	0.411
DREAM4 *in silico* size100										
GreyNet	**0.822**	**0.258**	**0.725**	**0.160**	**0.771**	**0.267**	0.731	**0.205**	**0.789**	**0.221**
BTNET(GB)	0.776	0.186	0.694	0.113	0.759	0.235	0.723	0.143	0.758	0.165
BTNET(AB)	0.776	0.207	0.699	0.116	0.770	0.224	0.740	0.158	0.780	0.169
SWING-RF	0.793	0.192	0.723	0.116	0.759	0.214	0.742	0.193	0.775	0.160
SWING-Dionesus	0.772	0.124	0.700	0.095	0.709	0.194	0.727	0.187	0.771	0.143
BiXGBoost	0.744	0.138	0.682	0.075	0.716	0.119	0.702	0.106	0.728	0.090
GENIE3-lag	0.790	0.167	0.711	0.103	0.767	0.215	**0.742**	0.152	0.786	0.146
Jump3	0.724	0.099	0.623	0.057	0.696	0.077	0.662	0.072	0.696	0.074
TIGRESS	0.715	0.054	0.532	0.037	0.483	0.018	0.467	0.018	0.521	0.022

The highest AUROC and AUPR are shown in bold for each network.

### 3.4 Performance on hepatocellular carcinoma data

Hepatocellular carcinoma (HCC) accounts for >90% of liver cancers with a five-year survival of only 18 % and the fourth leading cause of cancer-related deaths (Yang et al., 2019b; Villanueva, 2019). It is estimated that more than one million individuals will be affected by liver cancer annually by 2025 and the World Health Organization (WHO) predicted that the mortality of liver cancer will also arrive at one million in 2030 (Yang et al., 2019b; Villanueva, 2019; Llovet et al., 2021). It is imperative to search for important biomarkers of molecular and immune classes to guide therapy. A GRN of HCC will significantly benefit this kind of search.

**FIGURE 4:**
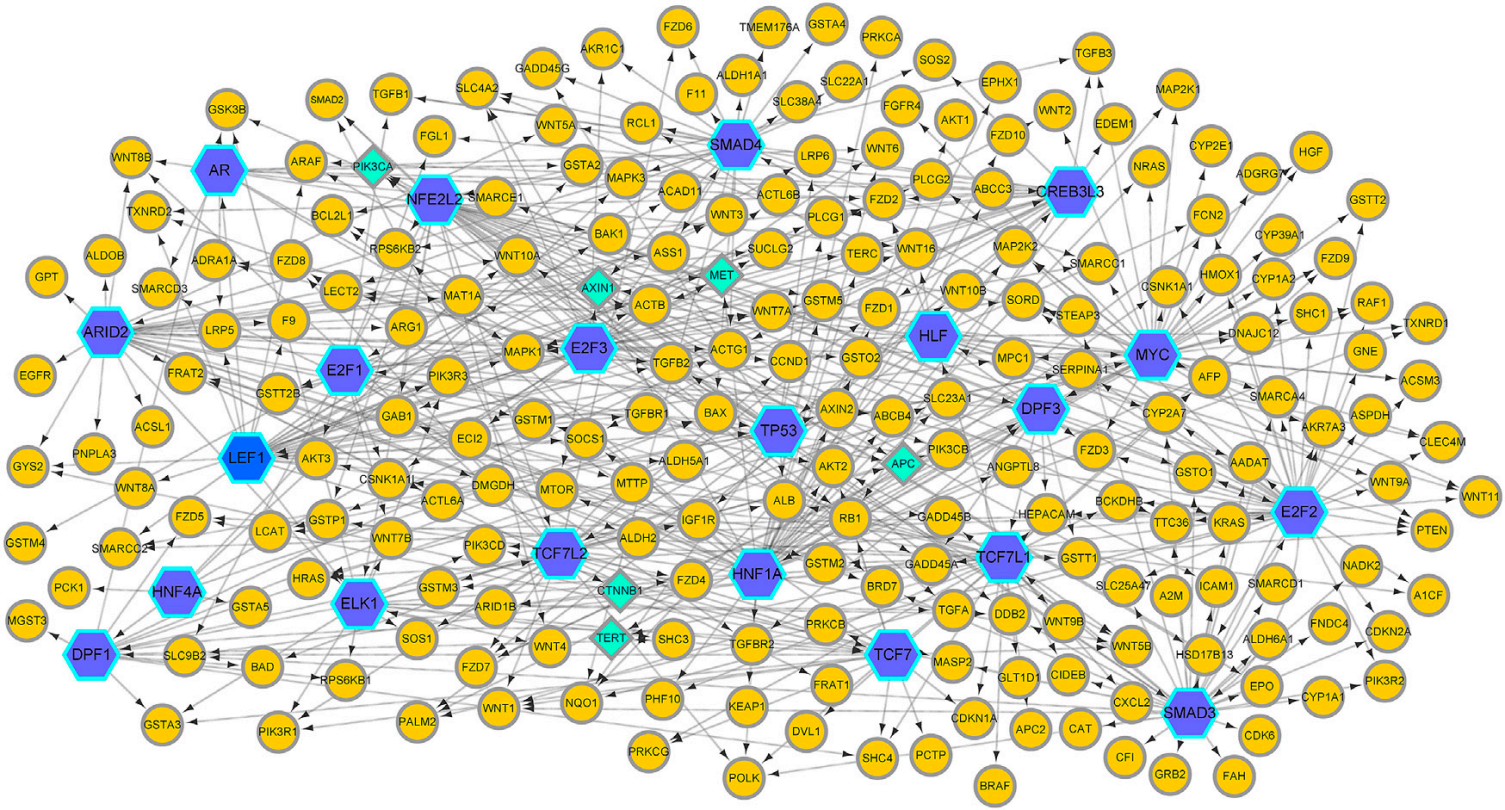
The HCC GRN reconstructed by GreyNet. In the network, the larger blue hexagon nodes represent TFs. The circle orange nodes represent the target genes. The diamond green nodes represent some elite disease genes in HCC.

In this article, we apply GreyNet to the HCC time-course expression data. We select the top 500 regulatory links in HCC GRN by the score of the inference. The inferred HCC GRN is shown in Figure 4. From Figure 4, we can see that, TP53, PIK3CA, AXIN1, MET, APC, CTNNB1, and TERT (all aforementioned genes are protein-coding genes) are elite genes highly related to HCC. TP53, TERT (promoter), and CTNNB1 are dominant mutational driver cancer genes, which account for 21–31 %, 44–65 %, and 27–40 % of patients with HCC (Yang et al., 2019b; Llovet et al., 2021). In terms of TF genes, ARID2 is correlated with the initiation and progression of HCC(Schulze et al., 2015); NFE2L2 is involved in hepatocarcinogenesis and progression (Nault et al., 2014; Niu et al., 2016); HNF1A is related to promoting genetic liver adenomatosis occurrence and possibly further malignant transformation to HCC(Zucman-Rossi et al., 2015).

Then, we enrich the HCC GRN by NOA (Wang et al., 2011). The result of the enriched Gene Ontology (GO) biological process and documented pathways in KEGG are shown in Table 4. From Table 4, we can see that the deregulations of the multiple signal pathways in HCC affect cell proliferation, RNA, nucleobase, nucleoside, nucleotide, nucleic acid metabolic process, and liver development. The ‘Wnt signaling pathway’ has a significant impact on cancer development and cancer mechanism evolution (Polakis, 2012; Zhan et al., 2017). The dysregulation of Wnt−/−*β*-catenin (a key component of the Wnt pathway) brings out the aberrant activation of signaling in HCC (Waisberg and Saba, 2015). Activated *β*-catenin translocates to the nucleus, interacting with TCF (T cell factor) and LEF (lymphoid enhancer-binding factor), and activates the transcription of the target genes which participate in CSC maintenance and EMT (Zhan et al., 2017; Farzaneh et al., 2021). Ultimately, it will lead to cell proliferation, angiogenesis, and anti-apoptosis. The “JNK pathway” is implicated in multiple cancers, including the regulation of liver tumorigenesis. In the mice model, it is shown that the increased expression of p21 (a cell-cycle inhibitor) can cause impaired proliferation. In human HCC, the activity of JNK can affect liver cell proliferation *via* p21 and c-Myc (a negative regulator of p21). It is found that the growth of xenografted human HCC cells can be reduced by pharmacologic inhibition of JNK (Hui et al., 2008; Dimri and Satyanarayana, 2020). The ‘receptor tyrosine kinase pathways’ implicate in activating multiple downstream signals, including the epidermal growth factor (EGF) receptor, the fibroblast growth factor (FGF) receptor, the hepatocyte growth factor (HGF/c-MET), the stem cell growth factor receptor c-KIT, the platelet-derived growth factor (PDGF) receptor, and the vascular endothelial growth factor (VEGF) receptor (Dimri and Satyanarayana, 2020). The “transforming growth factor-beta” (TGF-*β*) involves multiple stages of HCC development from liver injury toward fibrosis, cirrhosis, and cancer. In hepatocarcinogenesis, TGF-*β* performs as a suppressor factor in the early stages. However, TGF-*β* contributes to tumor progression latterly (Fabregat and Caballero-Díaz, 2018). The consistency between these enriched functions and the prior knowledge about HCC implies the effectiveness of GreyNet.

**TABLE 4:** The enrichment of GO biological process and KEGG pathway in HCC GRN.

GO:Term	Term Name	Representative Gene	Corr. *p*-val
GO:0016055	Wnt receptor signaling pathway	DVL1; WNT7B; DVL3; WNT8A; CCND1; WNT11; TCF7L2; FZD3; TCF7; LRP5; WNT9B; FZD1; WNT3; TCF7L1; FZD6; APC2; GSK3B; FRAT1; WNT4; FZD2; FZD4; APC; WNT5B; WNT7A; AXIN2; DVL2; CTNNB1; WNT1; CSNK1A1; FZD9; WNT10A; LEF1; FZD8; FRAT2; FZD5; WNT10B; AXIN1; WNT16; WNT5A	1.1E-54
GO:0008283	Cell proliferation	WNT7B; CCND1; BAD; FZD3; SMAD4; FZD6; GSK3B; WNT4; BAK1; BCL2L1; CTNNB1; TGFB1; WNT1; FZD9; BAX; TERC; MAP2K1; HGF; WNT10B; MET; WNT5A	7.0E-17
GO:0007169	Transmembrane receptor protein tyrosine kinase signaling pathway	AKT1; GRB2; IGF1R; PIK3R1; SOS1; SMARCC1; EGFR; PIK3R3; PTEN; FGFR4; GAB1; RAF1; HGF; MET	5.3E-10
GO:0051252	Regulation of RNA metabolic process	ARID2; HNF1A; AKT2; TCF7L2; TCF7; SMAD3; ELK1; TCF7L1; SMAD4; SMARCC1; GSK3B; E2F3; WNT4; TGFB3; RB1; CDKN2A; WNT7A; CTNNB1; TGFB1; WNT1; E2F2; EPO; LEF1; MAP2K1; SMAD2; WNT10B; AXIN1; MET	7.6E-9
GO:0043408	Regulation of MAPKKK cascade	AKT1; WNT7B; GRB2; AKT2; IGF1R; BRAF; APC; WNT7A; CTNNB1; GAB1; AXIN1; WNT5A	5.7E-9
GO:0006357	Regulation of transcription from RNA polymerase II promoter	ARID2; HNF1A; AKT2; TCF7L2; SMAD3; TCF7L1; SMAD4; SMARCC1; TGFB3; RB1; CTNNB1; TGFB1; WNT1; EPO; LEF1; MAP2K1; SMAD2; WNT10B; AXIN1; MET	1.7E-7
GO:0007265	Ras protein signal transduction	SHC3; GRB2; SOS1; CDKN1A; RB1; TP53; NRAS; MAPK3; RAF1; MAP2K1; CDKN2A; MAP2K2; KRAS; SHC2	3.3E-7
GO:0043405	Regulation of MAP kinase activity	WNT7B; GSK3B; TGFB3; GAB1; MAP2K1; PRKCA; HGF; AXIN1; MET; WNT5A	1.0E-7
GO:0006338	Chromatin remodeling	HNF1A; RB1; ACTL6A; ARID1A; SMARCC1; SMARCD1; ARID1B; SMARCC2; SMARCA2; SMARCB1	5.6E-6
GO:0046328	Regulation of JNK cascade	AKT1; WNT7B; AKT2; WNT7A; GAB1; AXIN1; WNT5A	2.3E-5
GO:0007179	Transforming growth factor beta receptor signaling pathway	SMAD4; SMAD3; TGFB1; TGFBR1; TGFB3; TGFBR2; TGFB2; SMAD2	9.1E-4
GO:0006139	Nucleobase, nucleoside, nucleotide and nucleic acid metabolic process	DPF1; HNF1A; NRAS; TCF7L2; DDB2; TCF7; SMARCD3; SMAD3; ELK1; TCF7L1; SMAD4; ABCC3; SMARCC1; KEAP1; E2F3; ACTL6A; ACTL6B; ARID1A; RB1; CDKN2A; CTNNB1; E2F2; TERT; PRKCB; POLK; UGP2; LEF1; TERC; SMAD2; SMARCC2; SMARCB1; AXIN1; DPF3	8.2E-4
GO:0007264	Small GTPase mediated signal transduction	SHC3; GRB2; SOS1; CDKN1A; RB1; TP53; BRAF; NRAS; MAPK3; RAF1; MAP2K1; SOS2; CDKN2A; MAP2K2; HMOX1; KRAS; SHC2	4.6E-4
GO:0043491	Protein kinase B signaling cascade	AKT1; RPS6KB1; AKT2; RPS6KB2	3.2E-4
GO:0001889	Liver development	CCND1; HGF; SMAD3; AFP; CTNNB1; HNF1A; ALDH2; ALDOB; ASS1	2.0E-4

## 4 Discussion

Limited by current technology and knowledge, the underlying gene regulatory mechanisms in cells are not very clear to us. It is reasonable to assume that gene interactions behave as a grey system. Moreover, the similarity and association of the two components are variational, evolving the time in the biological systems. It is less useful to assign a single static score fraction to two variables over an entire time-series. In this condition, we turned the static coefficient to the dynamic grey association by incorporating the adaptive sliding window technique to capture the dynamic evolution, which is much more aligned with real and known gene regulations.

The dynamic grey association is not enough to mine the causality in GRN. Thus, we further introduced both linear and non-linear regression methods to search for causal links by temporal information. Causal relationships between the variables can disclose the origin of the outcome and contribute to decision making. The Granger causal regression model is easy to be understood and explained.

Reconstructing the causal gene regulatory network is a preliminary step to finding out the internal mechanism of the biological procedure and facilitating our understanding of the basic pathology of tumors and other diseases (Lesage et al., 2018; Femerling et al., 2022). However, current biological datasets generated by the facilities are usually accompanied by a low rate of signal-to-noise ratio. Simultaneously, GRN inference is an ill-posed problem with sparsity. A purely data-driven model will find it hard to accurately find real and key regulatory links (Santra, 2014). Fortunately, many databases (Liu et al., 2015; Fang et al., 2020; Zhang et al., 2020) have been established to provide adequate regulatory prior knowledge. Fusing prior knowledge in the model may be an anticipated solution to improve the quality of the GRN topology (Ideker et al., 2011; Abdelzaher et al., 2015; Dandan et al., 2020). It is expected to investigate their effects on GreyNet in the future.

## 5 Conclusion

In this article, we proposed an interpretable machine-learning framework to infer the gene regulatory network from time-series expression data. We applied grey theory with the adaptive sliding window technique to model internal interactions in real regulatory procedures in the condition of limited information and knowledge. We further incorporated the Granger causality framework to search for causal regulations between genes. In DREAM4 *in silico* datasets, our model got competitive performances on AUROC and AUPRC compared with other state-of-the-art models. In the real HCC dataset, GreyNet can find meaningful pathways in HCC development from the functional enrichment results of HCC GRN. In the future, we will provide an update to make our model applicable to single-cell RNA-seq data.

## Data Availability

The original contributions presented in the study are publicly available. The data and code can be found here: https://github.com/zpliulab/GreyNet.
