# Supplementary material for: Inferring causal gene regulatory network via GreyNet: From dynamic grey association to causation
Source: Front Bioeng Biotechnol. 2022 Sep 27;10:954610. doi: 10.3389/fbioe.2022.954610 (PMC9551017; doi:10.3389/fbioe.2022.954610)
Supplement: Supplementary file 1 [file DataSheet1.zip › Supplementary File/Supplementary Material.pdf]

Supplementary Material

1 IMPLEMENTATION OF GREYNET

GreyNet applies dynamic grey association and Granger causality framework to reconstruct GRN from gene expression time series data. We firstly apply adaptive sliding window to capture dynamic temporal information. The default window length is 15 and the time lag is 1. Then, we introduce four different regression models to find causal and directional regulatory links. Hyperparameters of each regression model are selected by the grid search method. We set GreyNet-Xgboost as our default regression model. The default learning rate is 0.0001. The number of trees is set to 1000. The parameter $subsample$ is 0.8 which controls the ratio of samples in subtrees. The parameters $colsample\_bytree$ and $colsample\_bylevel$ are both set to 0.6, controlling the ratio of gene and each splitting node.

1.1 Figures

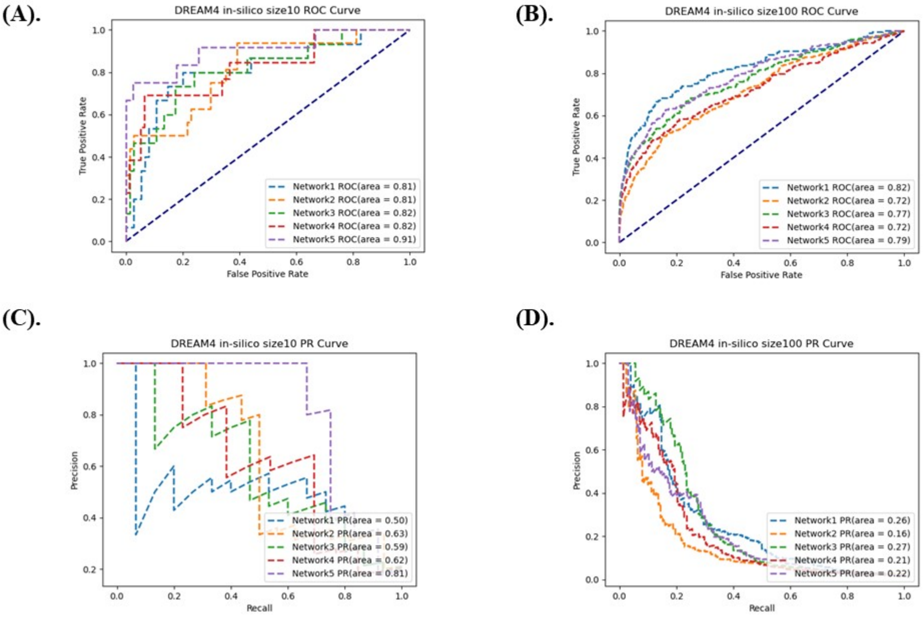

Figure S1. ROC and PR curves of GreyNet on DREAM4 in-silico datasets. (A) ROC curve on DREAM4 in-silico size10. (B) ROC curve on DREAM4 in-silico size100. (C) PR curve on DREAM4 in-silico size10. (D) PR curve on DREAM4 in-silico size100.

1.2 Tables

Table S1. The performances of GreyNet on DREAM4 in-silico dataset.

	AUROC	AUPRC	Accuracy	Recall	Precision	F1_score	MCC
DREAM4 in-silico size10							
Network1	0.825	0.440	0.833	0.793	0.471	0.590	0.522
Network2	0.819	0.577	0.697	0.919	0.339	0.494	0.426
Network3	0.827	0.520	0.788	0.795	0.402	0.531	0.456
Network4	0.841	0.514	0.885	0.702	0.562	0.619	0.562
Network5	0.905	0.743	0.919	0.784	0.685	0.715	0.683
DREAM4 in-silico size100							
Network1	0.819	0.249	0.860	0.679	0.082	0.147	0.202
Network2	0.723	0.154	0.821	0.521	0.073	0.128	0.143
Network3	0.765	0.258	0.756	0.664	0.052	0.096	0.135
Network4	0.727	0.199	0.820	0.551	0.065	0.115	0.141
Network5	0.790	0.210	0.825	0.627	0.068	0.122	0.164

Table S2. The AUROC performances on DREAM4 in-silico size100 data.

Methods	Network1	Network2	Network3	Network4	Network5	Avg AUROC	Std AUROC
BTNET(GB)	0.78	0.69	0.76	0.72	0.76	0.74	0.03
BTNET(AB)	0.78	0.70	0.77	0.74	0.78	0.75	0.03
SWING-RF	0.79	0.72	0.76	0.74	0.78	0.76	0.03
SWING-Dionesus	0.77	0.70	0.71	0.73	0.77	0.74	0.03
BiXGBoost	0.74	0.68	0.72	0.70	0.73	0.71	0.02
GENIE3-lag	0.79	0.71	0.77	0.74	0.79	0.76	0.03
Jump3	0.72	0.62	0.70	0.66	0.70	0.68	0.04
TIGRESS	0.72	0.53	0.48	0.47	0.52	0.54	0.10
BETS	0.78	0.65	0.64	0.70	0.67	0.69	0.06
GreyNet-LASSO	0.71	0.62	0.66	0.66	0.63	0.66	0.04
GreyNet-Ridge	0.77	0.65	0.72	0.72	0.73	0.72	0.04
GreyNet-RF	0.80	0.72	0.77	0.75	0.79	0.77	0.03
GreyNet-Xgboost	0.82	0.72	0.77	0.73	0.79	0.77	0.04

Table S3. The AUPRC performances on DREAM4 in-silico size100 data.

Methods	Network1	Network2	Network3	Network4	Network5	Avg AUPRC	Std AUPRC
BTNET(GB)	0.19	0.11	0.24	0.14	0.16	0.17	0.04
BTNET(AB)	0.21	0.12	0.22	0.16	0.17	0.17	0.04
SWING-RF	0.19	0.12	0.21	0.19	0.16	0.18	0.03
SWING-Dionesus	0.12	0.10	0.19	0.19	0.14	0.15	0.04
BiXGBoost	0.14	0.08	0.12	0.11	0.09	0.11	0.02
GENIE3-lag	0.17	0.10	0.22	0.15	0.15	0.16	0.04
Jump3	0.10	0.06	0.08	0.07	0.07	0.08	0.01
TIGRESS	0.05	0.04	0.02	0.02	0.02	0.03	0.01
BETS	0.16	0.10	0.13	0.14	0.11	0.13	0.02
GreyNet-LASSO	0.25	0.10	0.17	0.22	0.11	0.17	0.06
GreyNet-Ridge	0.19	0.09	0.17	0.18	0.11	0.15	0.04
GreyNet-RF	0.17	0.10	0.22	0.15	0.15	0.16	0.04
GreyNet-Xgboost	0.26	0.16	0.27	0.20	0.22	0.22	0.04
