# Supplementary figures and images for: Inferring causal gene regulatory network via GreyNet: From dynamic grey association to causation
Source: Front Bioeng Biotechnol. 2022 Sep 27;10:954610. doi: 10.3389/fbioe.2022.954610 (PMC9551017; doi:10.3389/fbioe.2022.954610)

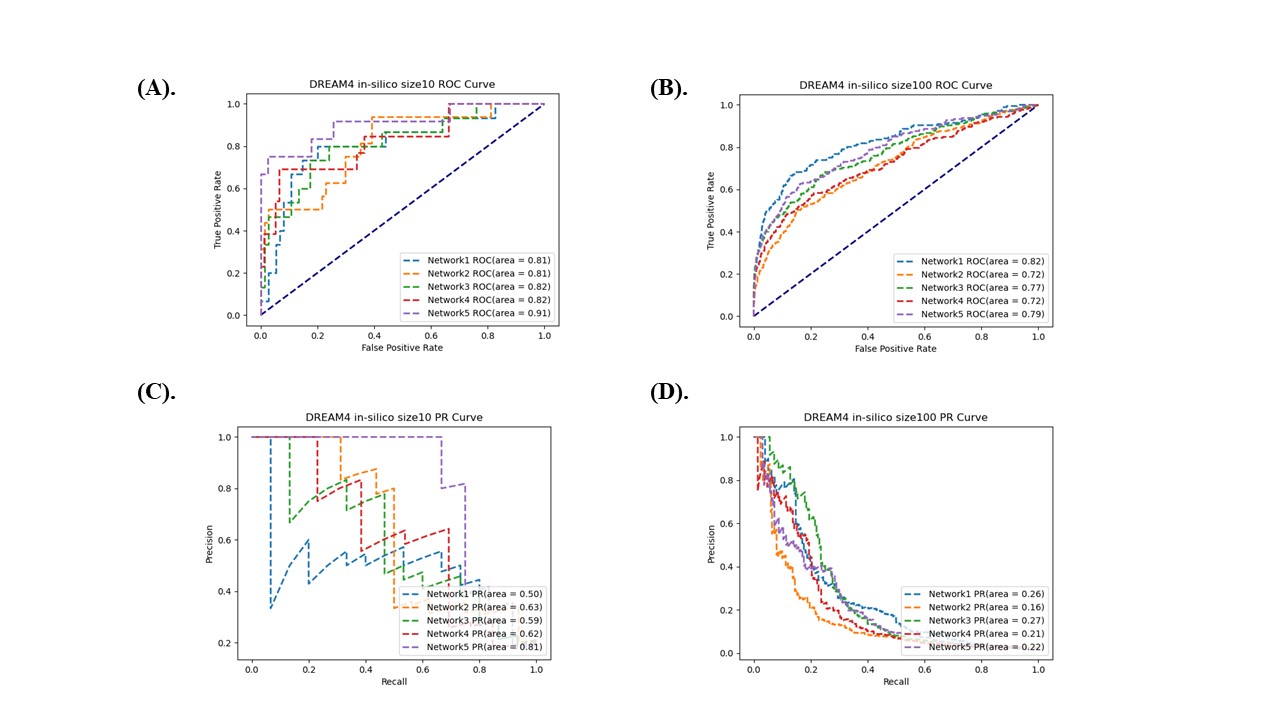

Supplement: Supplementary file 1 [file DataSheet1.zip › Supplementary File/FigS1.jpg]
